# Human carcinoma-associated mesenchymal stem cells promote ovarian cancer chemotherapy resistance *via* a BMP4/HH signaling loop

**DOI:** 10.18632/oncotarget.6870

**Published:** 2016-01-09

**Authors:** Lan G. Coffman, Yun-Jung Choi, Karen McLean, Benjamin L. Allen, Marina Pasca di Magliano, Ronald J. Buckanovich

**Affiliations:** ^1^ Division of Hematology Oncology, Department of Internal Medicine, University of Michigan Medical Center, Ann Arbor, Michigan, USA; ^2^ Division of Gynecologic Oncology, Department of Obstetrics and Gynecology, University of Michigan Medical Center, Ann Arbor, Michigan, USA; ^3^ Department of Cell and Developmental Biology, University of Michigan Medical Center, Ann Arbor, Michigan, USA; ^4^ Department of Surgery, University of Michigan Medical Center, Ann Arbor, Michigan, USA

**Keywords:** ovarian cancer, mesenchymal stem cell, tumor microenvironment, BMP4, Hedgehog

## Abstract

The tumor microenvironment is critical to cancer growth and therapy resistance. We previously characterized human ovarian carcinoma-associated mesenchymal stem cells (CA-MSCs). CA-MSCs are multi-potent cells that can differentiate into tumor microenvironment components including fibroblasts, myofibroblasts and adipocytes. We previously reported CA-MSCs, compared to normal MSCs, express high levels of BMP proteins and promote tumor growth by increasing numbers of cancer stem-like cells (CSCs). We demonstrate here that ovarian tumor cell-secreted Hedgehog (HH) induces CA-MSC *BMP4* expression. CA-MSC-derived BMP4 reciprocally increases ovarian tumor cell *HH* expression indicating a positive feedback loop. Interruption of this loop with a HH pathway inhibitor or BMP4 blocking antibody decreases CA-MSC-derived BMP4 and tumor-derived HH preventing enrichment of CSCs and reversing chemotherapy resistance. The impact of HH inhibition was only seen in CA-MSC-containing tumors, indicating the importance of a humanized stroma. These results are reciprocal to findings in pancreatic and bladder cancer, suggesting HH signaling effects are tumor tissue specific warranting careful investigation in each tumor type. Collectively, we define a critical positive feedback loop between CA-MSC-derived BMP4 and ovarian tumor cell-secreted HH and present evidence for the further investigation of HH as a clinical target in ovarian cancer.

## INTRODUCTION

Ovarian cancer is the most deadly US gynecologic malignancy, killing over 14,000 women yearly [1]. Most ovarian cancer cases are diagnosed as late stage disease with diffuse peritoneal metastasis indicating an ovarian cancer tropism for the intra-abdominal microenvironment. Despite initial chemotherapeutic response, most ovarian cancer patients will develop recurrent disease. Inevitably, recurrent disease will become resistant to standard platinum-based chemotherapy ultimately leading to patient death. The tumor microenvironment is critical for the growth, spread and survival of cancer cells with emerging data describing the importance of stromal components such as adipocytes and cancer associated fibroblasts in ovarian cancer [2-5].

Mesenchymal stem cells (MSCs) are an important component of the tumor microenvironment [6, 7]. MSCs were first characterized from bone marrow, adipose and embryonic tissues and are well-documented to travel to tumor sites [8-10]. MSCs have now been reported in a variety of other tissues including ovary, brain, spleen, liver, kidney, lung, muscle, thymus, pancreas, eyelid, and the peri-vasculature [11-13], suggesting MSCs may be present in many tissues throughout tumor initiation and growth.

Recently our lab isolated primary human ovarian carcinoma-associated MSCs (CA-MSCs) [14]. CA-MSCs are distinct from cancer associated fibroblasts; CA-MSCs meet all the established criteria for MSCs [15] with appropriate surface marker expression and multipotent differentiation potential. As a multi-potent stem cell population, CA-MSCs (unlike cancer associated fibroblasts) will passage for many months in culture. CA-MSCs have a normal genome and do not form tumors [16]. However, CA-MSCs are strongly pro-tumorigenic, increasing both tumor growth and “stemness” [14]. Importantly, as CA-MSCs are multi-potent cells capable of differentiating into several critical components of the tumor stroma including fibroblasts, osteocytes and adipocytes [15], therapeutic targeting of CA-MSCs could impact multiple components of the tumor microenvironment and thereby have a powerful anti-tumor effect.

CA-MSCs have a unique expression profile compared to normal adipose, bone marrow or ovary derived MSCs characterized by increased *BMP* expression (particularly *BMP2* and *BMP4*) [14, 16]. The factors leading to the unique expression profile of CA-MSCs are unknown; however our previous work suggests that tumor-secreted factors contribute to the CA-MSC expression profile.

One potential tumor derived CA-MSC regulating factor is Hedgehog (HH). HH signaling, which is critical during development and in maintenance of the adult stem cell pool, is a key regulator of BMP expression [17]. HH signaling also has emerging roles in tumor/stromal signaling [18]. In ovarian cancer, increased HH signaling is correlated with poor outcomes [19] and HH signaling is increased in ovarian cancer at the time of disease recurrence [20]. HH appears to be particularly important in the tumor microenvironment as HH signaling in ovarian tumor stroma is associated with enhanced chemoresistance and decreased survival [3, 19, 21]. In contrast, HH signaling appears to restrict tumor progression in bladder cancer and HH inhibition produced negative clinical results in pancreatic cancer. These contrasting results highlight the complexity of the HH pathway and may indicate tissue specific effects of HH signaling which would be unsurprising given the divergent effects of HH in spatiotemporal gradients during development [22, 23].

We investigated a possible role for HH signaling in CA-MSCs. We demonstrate that tumor-derived HH drives CA-MSC BMP4 production. CA-MSC-derived BMP4 reciprocally increases HH production in ovarian cancer cells thus creating a positive feedback loop between the tumor and CA-MSCs. This feedback loop promoted chemotherapy resistance both *in vitro* and *in vivo*. *In vivo* pharmacologic HH inhibition abrogated the pro-tumorigenic effects of CA-MSCs preventing increases in cancer stem cell-like cell (CSC) percentage and reversed chemotherapy resistance indicating that HH signaling is critical for the tumor growth promoting function of CA-MSCs.

## RESULTS

### Hedgehog signaling is active in the stroma of normal ovary and ovarian cancer

**Figure 1:**
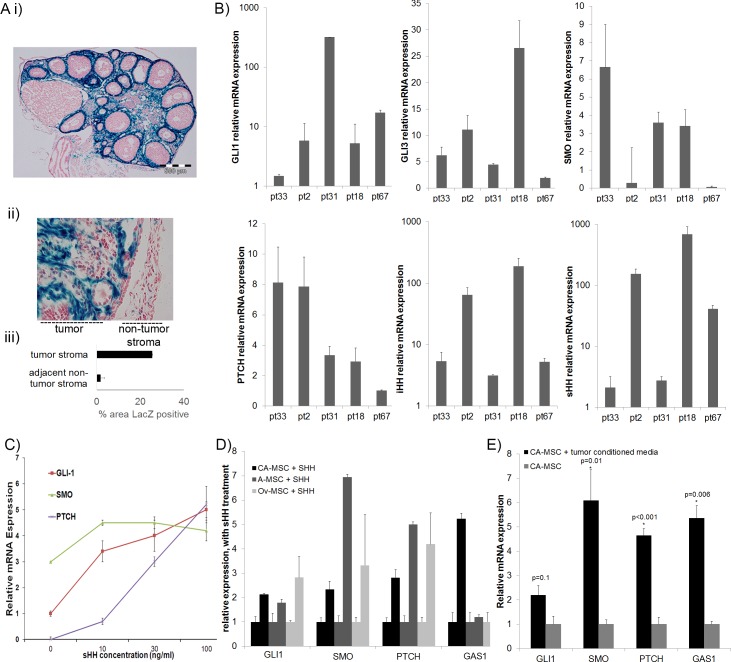
HH signaling is active in the normal ovary, ovarian tumor stroma and in MSCs **A.** Gli1-LacZ reporter mice demonstrate Gli1 expression (blue) in i) normal ovary stroma, and ii) ID8 ovarian tumor stroma with iii) quantification of Gli1-LacZ positive area in tumor stroma vs non-tumor stroma demonstrating significantly higher levels in tumor stroma (quantification via ImageJ analysis in 3 tumor and non-tumor sections). **B.** qRT-PCR analysis of GLI1, GLI3, SMO, PTCH, IHH and SHH in primary ovarian tumors confirming HH signaling components are expressed in all tumors tested. **C.** SHH treatment of normal adipose derived MSCs demonstrate dose dependent activation of the canonical HH signaling pathway (data are normalized to untreated adipose MSC GLI1 value). **D.** qRT-PCR demonstrating treatment of A-MSCs, normal ovary (Ov-MSCs) and CA-MSCs with recombinant SHH activates HH signaling pathway. **E.** qRT-PCR demonstrating tumor conditioned media (TCM) likewise activates HH signaling in CA-MSCs. Error bars=standard error of the mean.

To explore the role of HH signaling in the ovarian cancer microenvironment we first confirmed HH signaling in normal ovarian tissue and ovarian tumors. To confirm HH activity in normal ovaries and ovarian tumors we used a *Gli1^lacZ^* reporter mouse [24, 25]. Gli1 is both a downstream component of HH signaling and a transcriptional target, thus its expression indicates pathway activation [26]. We observed strong Beta-Galactosidase (β-Gal) activity throughout the normal murine ovarian stroma (Figure 1Ai). β-Gal expression was not observed in the ovarian surface epithelium, in developing follicles, or in the epithelial lining of the oviduct (the murine equivalent of the fallopian tube). β-Gal expression was detected in the peri-vasculature; a reported location for tissue associated MSCs [12].

To determine if HH signaling is active in ovarian tumor stroma, we transplanted ID8 mouse ovarian tumor cells into the flank of *Gli1^lacZ^* mice. β-Gal as an indicator of HH signaling was clearly noted within the tumor stroma with significantly less β-Gal in adjacent non-tumor stroma (Figure 1Aii, iii). To confirm HH signaling in human ovarian cancer, qRT-PCR of cDNA generated from primary human ovarian tumor samples were analyzed. Consistent with previous results [27], *GLI1* and *GLI3* (HH pathway transcriptional effectors), *PTCH1* (HH signaling repressor and target gene), *SMO* (HH signaling activator), *IHH* and *SHH* (HH pathway ligands) were expressed in ovarian tumors, albeit at variable levels (Figure 1B).

### Mesenchymal stem cells respond to HH ligands produced by ovarian cancer cells

Given the largely stromal localization of HH pathway activation, we next explored the ability of MSCs to respond to HH signaling. We tested the ability of both normal ovary derived MSCs (Ov-MSCs) and, given the predilection of ovarian cancer for omental adipose, normal adipose derived MSCs (A-MSCs) to respond to HH. A-MSCs and Ov-MSCs treated with recombinant Sonic Hedgehog (SHH) demonstrated increased expression of downstream targets of the canonical HH pathway indicating both MSC groups respond to HH signaling (Figure 1C, 1D). CA-MSCs also demonstrated clear response to HH treatment with induction of *GLI1*, *SMO*, *PTCH1* and *GAS1* (Figure 1D).

To determine if cancer cells are a source of HH ligands, we treated CA-MSCs with conditioned media from multiple ovarian cancer cell lines or primary human ovarian cancer cell cultures. The induction of HH responsive genes was analyzed via qRT-PCR. Tumor conditioned media (TCM) lead to a similar pattern of HH target gene induction as seen with recombinant SHH (Figure 1E). This suggests that ovarian cancer cells produce HH ligands that can activate HH signaling pathways in MSCs.

### Tumor-derived HH differentially induces the expression of BMP4 in CA-MSCs

**Figure 2:**
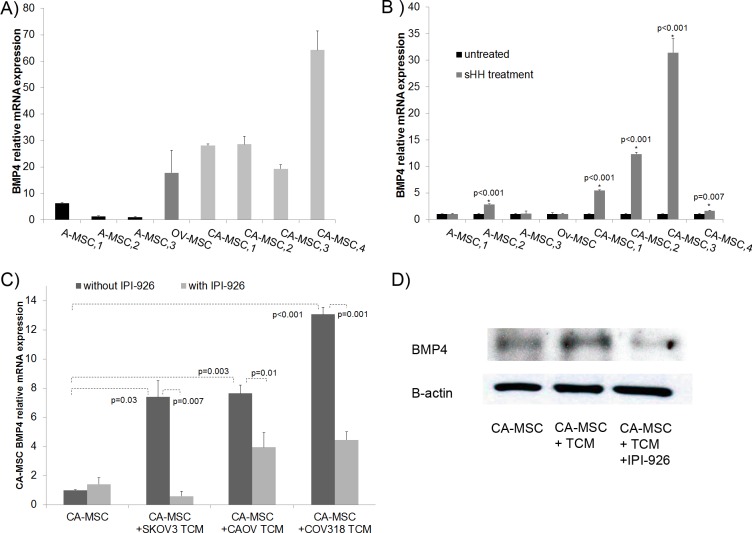
CA-MSCs respond to tumor derived HH with increased BMP4 **A.** qRT-PCR analysis of BMP4 expression in normal adipose MSCs (A-MSC1,2,3), normal ovary MSCs (Ov-MSC) and patient derived CA-MSCs (CA-MSC 1-4) demonstrating higher baseline BMP4 expression in CA-MSCs, relative expression compared to A-MSC3. **B.** qRT-PCR analysis of BMP4 expression in A-MSC, Ov-MSC and CA-MSCs without and with SHH demonstrating SHH-mediated BMP4 induction in CA-MSCs (expression is normalized to untreated controls for each pair). **C.** qRT-PCR analysis of BMP4 expression in CA-MSCs demonstrating that tumor conditioned media (TCM) derived from 3 ovarian cancer cell lines induces BMP4 expression and this induction can be blocked by the HH inhibitor IPI-926, all samples normalized to CA-MSC without IPI-926. **D.** Western blot of BMP4 protein levels in CA-MSCs ± TCM with or without IPI-926. Error bars=standard error of the mean.

Given (i) the responsiveness of MSCs to HH signaling, (ii) the role of HH in regulating *BMP* expression [17], and (iii) the differential expression of *BMP*s in CA-MSCs compared to normal controls, we assessed if HH activation could be the etiology of increased BMP signaling in CA-MSCs. qRT-PCR analysis confirmed greater baseline expression of *BMP2* and *BMP4* in CA-MSCs compared to normal Ov-MSCs and A-MSCs (data not shown and Figure 2A). HH treatment of A-MSCs and Ov-MSCs did not result in a significant (0-2.8 fold) induction of either *BMP2* or *BMP4* (Supplemental Figure 1 and Figure 2B). However, treatment of CA-MSCs with HH led to significant induction (5-30 fold) of *BMP4* (Figure 2B). *BMP2* expression was modestly increased in CA-MSCs (Supplemental Figure 1).

Given the HH-mediated induction of *BMP4* in CA-MSCs, we investigated if tumor derived HH could likewise induce *BMP4* expression. CA-MSCs were treated with TCM from three human ovarian cancer cell lines (SKOV3, COV318, CAOV-3). *BMP4* expression was increased in response to treatment with all three TCM (Figure 2C). Treatment with IPI-926, a SMO inhibitor, significantly decreased the TCM-mediated induction of *BMP4* transcription (Figure 2C) and BMP4 protein levels (Figure 2D) suggesting activation of *BMP4* expression is via tumor cell produced HH ligands.

### A paracrine HH-BMP4 ovarian cancer cell-CA-MSC positive feedback loop

**Figure 3:**
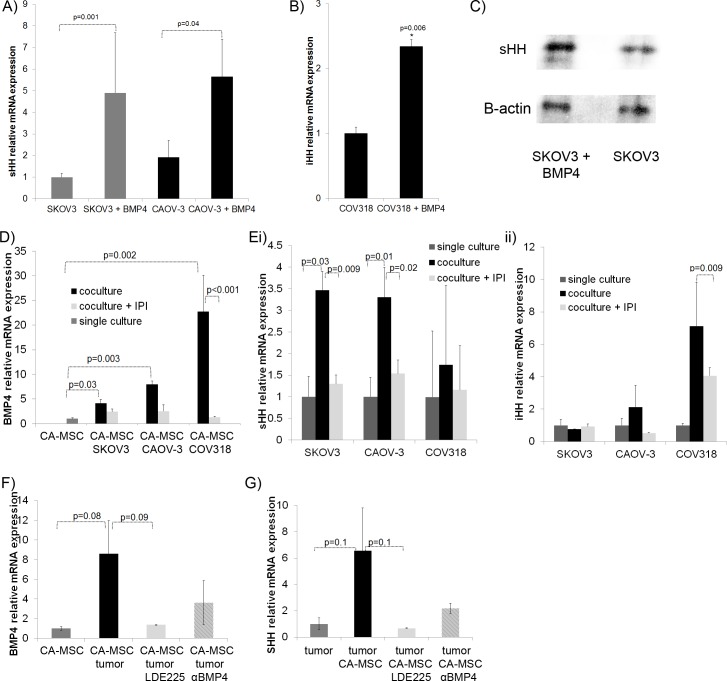
Ovarian tumor cells respond to BMP4 with increased HH forming a positive feedback loop interrupted by HH inhibition **A.** qRT-PCR analysis of ovarian cancer lines SKOV3 and CAOV-3 treated with BMP4 (200ng/ml) demonstrates induction of SHH. **B.** qRT-PCR analysis of ovarian cancer line COV318 treated with BMP4 demonstrates induction of IHH. **C.** Induction of SHH was verified at the protein level via western blot. **D.** qRT-PCR analysis of CA-MSCs grown in co-culture with tumor cell lines (SKOV3, CAOV-3 and COV318) for 5 days ± IPI-926 demonstrates BMP4 induction which is abrogated by IPI-926. **E.** i) qRT-PCR analysis of SKOV3 and CAOV-3 cells grown in co-culture with CA-MSCs demonstrate tumor SHH induction blocked by IPI-926, ii) qRT-PCR analysis of COV318 cells demonstrate IHH induction with CA-MSC co-culture decreased by IPI-926. **F.** qRT-PCR analysis of average BMP4 expression of CA-MSCs grown in co-culture with SKOV3 or CAOV-3 cells demonstrating decreased BMP4 induction with the SMO inhibitor LDE225 or a BMP4 blocking antibody. **G.** qRT-PCR analysis of average SHH induction of SKOV3 or CAOV-3 cells co-cultured with CA-MSCs demonstrating decreased SHH induction with LED225 or a BMP4 blocking antibody. Error bars=standard error of the mean.

We previously showed that CA-MSC-derived BMP directly impacts ovarian cancer cells [14]. We therefore hypothesized that CA-MSC-derived BMP4 may alter *HH* expression in tumor cells though a feedback mechanism. To investigate this possibility, we treated ovarian cancer cells with recombinant BMP4 and analyzed *SHH* expression via qRT-PCR. *SHH* expression was increased in SKOV3 and CAOV-3 cell lines after 48hrs of BMP4 treatment (Figure 3A). This induction was seen at both early (16hr) and later (48hr) time points (data not shown) indicating that BMP4 treatment is not selecting for a population of cells with higher *SHH* expression but inducing a general increase in *SHH* expression. In the COV318 ovarian cancer cell line, BMP4 treatment induced *IHH* rather than *SHH* (Figure 3B). *HH* induction was verified at the protein level via western blot (Figure 3C). The reciprocal induction of tumor derived *HH* by BMP4 creates a possible signaling loop with tumor derived HH and CA-MSC derived BMP4. To investigate the possibility of this positive feedback loop, we next utilized a transwell system for co-culture of CA-MSCs and ovarian tumor cells. The transwell membrane allowed only indirect cell/cell contact therefore interactions based on secreted factors were assessed. Cells were co-cultured for 5 days ± IPI-926. Cells were harvested from their respective chambers (CA-MSCs in the upper well, tumor cells in the lower well) and analyzed via qRT-PCR to determine levels of *BMP4* in CA-MSCs and *HH* in tumor cells. Co-culture resulted in clear induction of *BMP4* in CA-MSCs that was decreased in the presence of IPI-926 (Figure 3D). Likewise, SKOV3 and CAOV-3 tumor cells demonstrated significant induction of *SHH* while COV318 cells demonstrated significant induction of *IHH* (Figure 3E). IPI-926 diminished both *SHH* and *IHH* induction though effects on *IHH* induction were more modest (Figure 3Eii). IPI-926 treatment of CA-MSCs grown in single culture did not affect *BMP4* expression levels or cellular viability. Analogously, IPI-926 treatment of tumor cells grown alone had no effect on tumor cell *HH* expression or cellular viability (Supplemental Figure 2). Similarly, another SMO inhibitor, LDE-225, and a BMP4 blocking antibody were used in the co-culture system (with SKOV3 and CAOV-3 tumor cells) and both demonstrated a reduction in CA-MSC derived BMP4 and tumor derived HH (Figure 3F, 3G). These findings indicate a tumor/MSC HH/BMP4 signaling loop.

### Inhibition of HH signaling blocks the pro-tumorigenic effects of CA-MSCs

**Figure 4:**
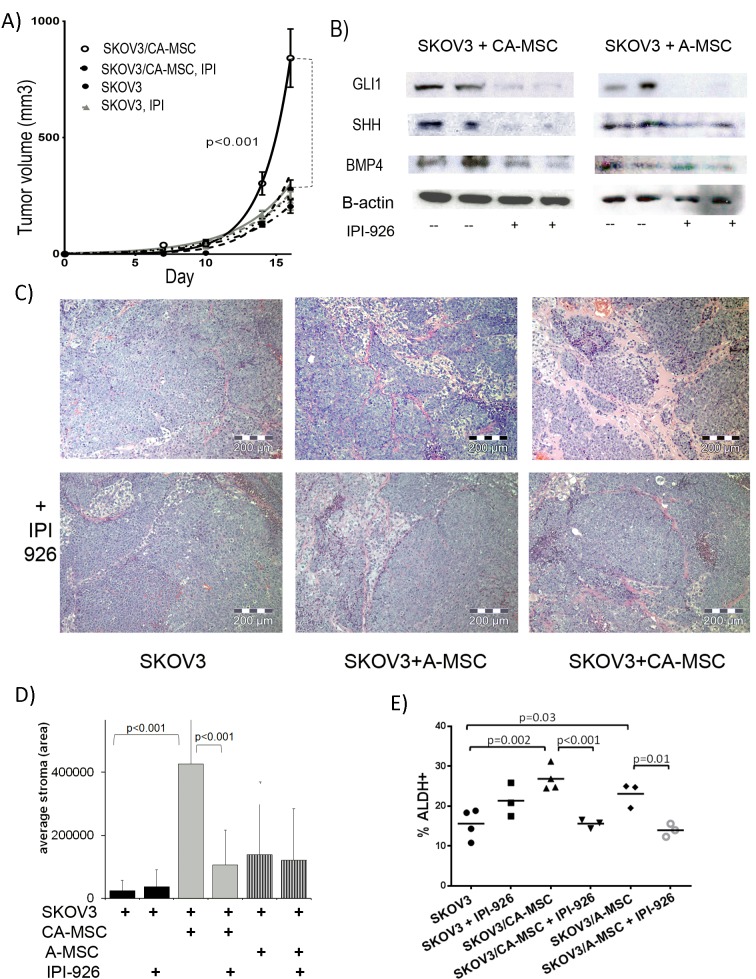
HH inhibition blocks CA-MSC mediated tumor growth promotion and stromal deposition **A.** Tumor growth curves of SKOV3 ovarian tumor cells ± CA-MSCs treated with and without IPI-926 demonstrating HH inhibition blocks CA-MSC-mediated increases in tumor growth. **B.** Western blot analysis of GLI1, SHH and BMP4 protein levels in SKOV3+CA-MSC and SKOV3+A-MSC tumors ± IPI-926 treatment confirm on-target effects of HH inhibition and subsequent decreases in BMP4 and SHH. **C.** Representative H&E stains of tumors: SKOV3, SKOV3+A-MSC or SKOV3+CA-MSC ± IPI-926 illustrating IPI-926 blocks MSC-mediated increases in stroma. **D.** Quantification of stroma in tumors (4 tumors per group, 8 low power sections per tumor). **E.** Percent ALDH+ cells from SKOV3 ± CA-MSCs or A-MSCs ± IPI-926 xenografts demonstrating IPI-926 blocks the CA-MSC-mediated increase in ALDH+ cells (each scatter plot represents an independently analyzed tumor, at least three tumors per group). Error bars=standard error of the mean.

We previously demonstrated that CA-MSCs significantly promote tumor growth in a BMP2/4-dependent manner. We therefore investigated whether disruption of the HH:BMP4 signaling loop with a HH inhibitor could alter the pro-tumorigenic effects of CA-MSCs. We created xenografts using SKOV3 cells alone, SKOV3 + A-MSCs and SKOV3 + CA-MSCs. Mice were treated with IPI-926 (20mg/kg daily via IP injection for 21 days) or vehicle control. Mice were sacrificed when tumor volumes reached size criteria or treatment was complete. Consistent with our previous report [14], untreated SKOV3+CA-MSC tumors grew significantly faster than SKOV3+A-MSC or SKOV3 alone tumors (Figure 4A and Supplemental Figure 3A). Surprisingly, IPI-926 treatment completely abolished the growth advantage provided by CA-MSCs (Figure 4A and Supplemental Figure 3B). IPI-926 had no effect on the growth of SKOV3 alone tumors, highlighting a critical role of human CA-MSCs in HH mediated tumorigenesis (Figure 4A).

Confirming on-target effects of IPI-926, western blot analysis demonstrated clear decreases in GLI1 levels in IPI-926 treated tumors consistent with inhibition of canonical HH signaling (Figure 4B). As predicted, BMP4 levels are highest in SKOV3+CA-MSC tumors. Supporting a positive feedback loop between tumor derived HH and CA-MSC derived BMP4, there was a 3-fold reduction in BMP4 protein levels in SKOV3+CA-MSC tumors treated with IPI-926 compared to SKOV3+CA-MSC untreated tumors (Figure 4B). Likewise, SHH protein levels were also reduced (4-fold) in SKOV3+CA-MSC tumors treated with IPI-926 compared to untreated tumors.

Histologic analysis of paraffin embedded tumors demonstrated significant differences in the amount of stroma in CA-MSC containing tumors (∼17 fold increase compared to SKOV3 alone tumors) (Figure 4C, 4D). This was also observed to a lesser extent with A-MSC containing tumors (∼5 fold increase). For CA-MSC containing tumors, treatment with IPI-926 significantly reduced the amount of tumor stroma (Figure 4C, 4D). This suggests that HH signaling affects MSC contribution to tumor stromal elements.

### HH inhibition blocks CA-MSC mediated increase in cancer stem cell-like cell percentage

We previously demonstrated that CA-MSCs (and to a lesser extent A-MSCs) increase the percentage of ALDH^+^ cancer stem cell-like cells (CSCs) [14, 28]. Evaluation of the tumor xenografts above demonstrated that, consistent with previous results, there is a significant increase in the percentage of ALDH^+^ cells in the SKOV3+CA-MSC tumors compared to SKOV3 alone tumors. This increase in ALDH^+^ cells is completely blocked with IPI-926 treatment (Figure 4E). SKOV3+A-MSC tumors also demonstrate a modest increase in the percentage of ALDH^+^ cells which is blocked with IPI-926 treatment (Figure 4E). IPI-926 treated SKOV3 alone tumors demonstrated no statistically significant change in the percentage of ALDH^+^ cells. The increase in ALDH^+^ cells is not primarily related to ALDH^+^ MSCs; when dsRed labeled MSCs were co-grown with SKOV3 cells, dsRed^+^ cells represented ∼1.2% of all ALDH^+^ cells thus MSC are not significantly impacting the observed differences in ALDH^+^ cells between groups (Supplemental Figure 3C).

### HH inhibition blocks CA-MSC induced chemotherapy resistance in ovarian tumor cells *in vitro* and *in vivo*

**Figure 5:**
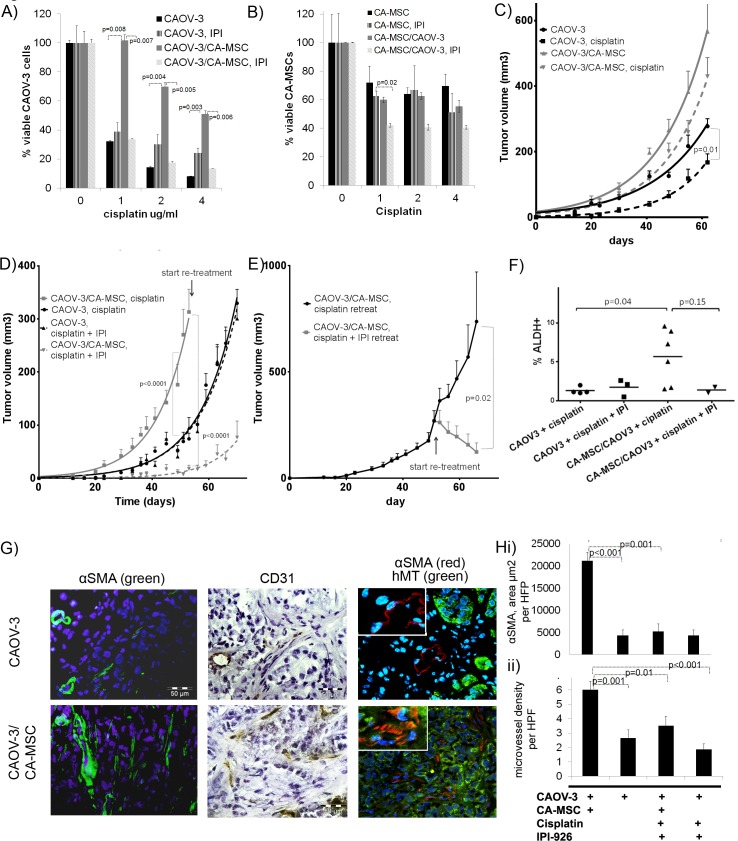
CA-MSCs promote chemotherapy resistance which can be reversed with HH inhibition **A.**-**B.** *In vitro* co-culture of GFP-CAOV-3 tumor cells ± CA-MSCs treated with cisplatin ± IPI-926, **A.** co-culture with CA-MSCs enhance tumor cell platinum-resistance and is reversed with IPI-926, graph represents % viable GFP-CAOV-3 cells normalized to 0ug/ml cisplatin group. **B.** CA-MSCs demonstrate cisplatin-resistance is not significantly altered by co-culture with tumor cells or co-treatment with cisplatin + IPI-926, graph represents % viable CA-MSCs normalized to 0ug/ml cisplatin group. **C.** Growth curve of CAOV-3 and CAOV-3/CA-MSC xenografts treated ± cisplatin demonstrating CA-MSCs promote platinum-resistance *in vivo* **D.** Growth curve of CAOV-3 and CAOV-3/CA-MSC xenografts treated with cisplatin ± IPI-926 demonstrating IPI-926 prevents CA-MSC-mediated platinum-resistance. **E.** Growth curve of CAOV-3/CA-MSC xenografts retreated with cisplatin ±IPI-926 starting on day 53 demonstrating IPI-926 reverses chemotherapy resistance in established tumors. **F.** Percent ALDH+ cells from CAOV-3±CA-MSCs xenografts treated with cisplatin±IPI-926 demonstrating IPI-926 blocks CA-MSC-mediated increases in ALDH+ cells. **G.** IF of CAOV-3 and CAOV-3/CA-MSC xenografts with anti-alpha smooth muscle actin (αSMA) (green), anti-αSMA (red) /anti-human mitochondrial antibody (hMT-green) demonstrating increased human stroma in CA-MSC-containing tumors and IHC of anti-CD31 (human/mouse). **H.** i) Quantification of αSMA per high power field (HPF) demonstrating IPI-926 blocks CA-MSC-mediated increases in fibroblasts ii) Quantification of microvessel density per HPF illustrating IPI-926 blocks CA-MSC-mediated increase in microvessel density. Error bars=standard error of the mean.

Given CA-MSCs can differentiate into distinct stromal cells (such as cancer associated fibroblasts and adipocytes) which promote chemotherapy resistance, and CA-MSCs increase ALDH+ CSCs which are chemotherapy resistant [28], we hypothesized that CA-MSCs would significantly enhance ovarian tumor chemotherapy resistance. We therefore investigated the role of CA-MSCs and the tumor/stromal HH/BMP4 signaling loop in resistance to platinum based chemotherapy, the core of ovarian cancer therapy. We treated GFP-labeled CAOV-3 cells grown in (i) direct co-culture with CA-MSCs or (ii) in single culture at equal densities and treated for 48 hours with increasing amounts of cisplatin. Viable cells were analyzed with FACS to distinguish GFP-labeled cancer cells from CA-MSCs. Co-culture of CAOV-3 cells with CA-MSCs, compared to CAOV-3 cells alone, resulted in 3-9 fold increases in tumor cell viability in response to cisplatin (Figure 5A). Addition of IPI-926 drastically decreased CA-MSC-mediated CAOV-3 cisplatin resistance while not significantly altering the efficacy of cisplatin in CAOV-3 cells grown in single culture (Figure 5A). In contrast, CA-MSCs grown in single culture demonstrated significant resistance to even high doses of chemotherapy (Figure 5B). The sensitivity of CA-MSCs to cisplatin was not significantly altered by co-culture with CAOV-3 cells or the addition of IPI-926 (Figure 5B). Interestingly, a modest decrease in CA-MSCs was seen with IPI-926 + cisplatin in CA-MSC/CAOV-3 co-culture. The ability of IPI-926 to block CA-MSC-mediated chemotherapy resistance was also replicated using co-culture of CA-MSCs and GFP-labeled PEO-1 and Hey1 ovarian cancer cell lines— lines which express both *SHH* and *IHH* (Supplemental Figure 4 and data not shown).

We next determined if the same effect on chemotherapy resistance was observed *in vivo*. CAOV-3 cells labeled with luciferase were grown ±CA-MSCs in the bilateral axilla of NOD/SCID mice and treated with cisplatin (1mg/kg weekly for 3 weeks) or vehicle control. As expected, cisplatin significantly reduced the growth of CAOV-3 tumors (Figure 5C). Once again the addition of CA-MSCs to CAOV-3 tumors significantly increased growth relative to CAOV-3 cells grown alone. Importantly, in the presence of CA-MSCs, CAOV-3 cells no longer demonstrated a significant response to cisplatin (Figure 5C). In fact CAOV-3/CA-MSC tumors treated with cisplatin demonstrated greater growth than untreated CAOV-3 cells grown alone (Figure 5C). To ensure changes in tumor volume were not due to overgrowth of the CA-MSC cells, bioluminescence data was also captured to assess the luciferase labeled CAOV-3 fraction. Bioluminescence measurements were consistent with tumor volume measurement indicating the change in tumor size corresponds with change in tumor cell volume (Supplemental Figure 5A).

To assess the impact of HH inhibition on CA-MSC-mediated chemotherapy resistance, CAOV-3 cells ± CA-MSCs in were injected into the bilateral axilla of NOD/SCID mice. Tumors were treated with cisplatin as described above (starting on day 7) ± daily treatment of IPI-926 (20mg/kg/day starting on day 5 after injection and continuing for 21 days). Cisplatin treated CAOV-3/CA-MSC tumors grew significantly faster than cisplatin treated CAOV-3 alone tumors. The addition of IPI-926 to cisplatin treatment of CAOV-3 alone tumors had no impact on tumor growth. However, IPI-926 with cisplatin lead to a striking decrease in the growth of CAOV-3/CA-MSC tumors with 2 out of 8 tumors failing to initiate (engraftment rates were 100% in all other groups) (Figure 5D).

To assess the impact of HH inhibition on established tumors, once tumors from mice bearing CAOV-3/CA-MSC tumors treated with cisplatin reached ∼300mm3 in volume, mice were randomized and then retreated with (i) cisplatin alone (1mg/kg weekly for up to 3 weeks) or (ii) cisplatin with IPI-926 (starting three days prior to the first cisplatin dose). Cisplatin treatment alone had no impact on tumor growth indicating chemotherapy resistance. In contrast, retreatment with cisplatin + IPI-926 lead to not only cessation in tumor growth but to tumor regression while tumors in mice retreated with cisplatin alone continued to grow rapidly (Figure 5E). This demonstrates that not only does blocking HH signaling lead to prevention of CA-MSC mediated chemotherapy resistance when tumors are small or just starting to engraft, but also significantly impacts tumors which have been firmly established and have been previously treated with chemotherapy.

Analysis of tumor ALDH expression was consistent with our previous findings with SKOV3 xenografts, demonstrating increases in the percentage of ALDH^+^ cells in CA-MSC containing tumors which is decreased in tumors treated with IPI-926 (Figure 5F). Histologic analysis demonstrates that CA-MSC containing tumors have significantly higher microvessel density (as determined through quantification of human and mouse CD31 positive vessels per high power field) (Figure 5G, 5Hii). IPI-926 treatment blocked this increase in blood vessel density. Likewise, CA-MSC containing tumors had significantly more alpha-smooth muscle actin (αSMA) positive cells than CAOV-3 cells alone and this effect was decreased with IPI-926 treatment (Figure 5G, 5Hi). Co-staining with an anti-human mitochondrial antibody (anti-hMt) demonstrated that the majority of αSMA positive cells within CA-MSC-containing tumors are of human origin and therefore likely represent differentiated CA-MSCs. αSMA positive cells within the CAOV-3 alone tumors did not co-stain with anti-hMt and are therefore likely of murine origin. Also of note, the staining pattern of αSMA in CAOV-3 alone tumors is largely perivascular and likely to represent vascular associated pericytes whereas αSMA positive cells in the CA-MSC containing tumors, in addition to being found perivascularly, were also present in large extra-vascular bands consistent with activated fibroblasts. This is consistent with our findings in SKOV3 xenografts which demonstrated clear, gross changes in the amount of tumor stroma in CA-MSC containing tumors which is reversed with HH inhibition.

Collectively, our results demonstrate that HH pathway inhibition prevents CA-MSC-mediated tumor growth promotion and enhancement of chemotherapy resistance. This points to a tumor/CA-MSC HH/BMP4 signaling loop as a critical mediator of the pro-tumorigenic effects of CA-MSCs.

## DISCUSSION

**Figure 6:**
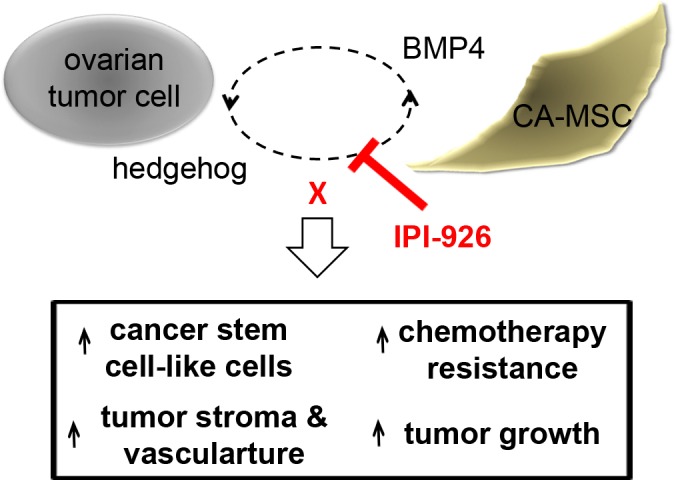
Schematic of ovarian tumor cell:CA-MSC hedgehog:BMP4 feed forward loop Ovarian tumor derived HH induces increased expression of BMP4 in CA-MSCs. CA-MSC derived BMP4 feeds back and induces increased expression of tumor derived HH. Blocking this signaling loop with the HH inhibitor IPI-926 prevents CA-MSC mediated increases in cancer stem cell-like cells, tumor growth, tumor stroma, tumor vasculature and chemotherapy resistance.

Increased HH signaling is correlated with poor outcomes in ovarian cancer [19]. Further, HH signaling is increased at the time of disease recurrence indicating the importance of this pathway in ovarian cancer [20]. Previous work indicates that HH acts primarily on the stroma [18]. However, which cells in the stroma HH acts upon was unclear. We now show that CA-MSCs are a critical target for stromal HH activation. HH activation of CA-MSCs results in the previously reported increased CA-MSC expression of *BMP4* [14]. BMP4 in turn increases tumor *HH* expression, thus creating a positive feedback loop (Figure 6). We find this loop is critical to CA-MSC-mediated increases in tumor growth and promotes chemotherapy resistance associated with increases in ALDH^+^ ovarian cancer stem-like cells. Interruption of this signaling loop with pharmacologic HH inhibition prevents the growth-promoting effects of CA-MSCs *in vivo*, reverses chemotherapy resistance and normalizes CSC percentages, indicating the importance of HH signaling to the pro-tumorigenic function of CA-MSCs. Our work is consistent with studies indicating HH inhibition can overcome stromal mediated chemotherapy resistance [3]. These prior studies found HH inhibition could overcome taxane but not platinum resistance. Platinum is the cornerstone of ovarian cancer therapy. Our work shows that HH inhibition can overcome platinum resistance even in well-established tumors.

### Incorporating CA-MSC in tumor xenografts

Our work is unique from the prior studies of HH as CA-MSCs create a ‘humanized’ tumor stroma. Unlike most ovarian cancer cell line xenografts and later passage PDX, CA-MSC containing tumors have a robust human stroma more analogous to that seen in patient tumors. This is important as we observed minimal effectiveness of HH inhibition, either alone or in combination with chemotherapy, in the standard human cell line xenograft model. However we observed robust response in tumors with humanized stroma. This indicates the importance of studying therapeutics in the context of a human stroma.

We previously demonstrated human fibroblast, adipocyte and osteoblast mRNA expression in xenografts containing human CA-MSCs. We now use IHC analysis with anti-human mitochondria antibodies to confirm CA-MSC containing xenografts have a predominantly human tumor stroma. CA-MSC containing xenografts have increased numbers of αSMA^+^ activated myofibroblasts which is prevented by HH inhibition. Additional studies will be necessary to determine the mechanism whereby HH inhibition reverses CA-MSC mediated stromal formation. It is interesting to note that CA-MSCs by themselves are highly chemotherapy resistant. Thus CA-MSCs are likely preserved following chemotherapy and could support the expansion and ultimately the drug resistance of residual cancer cells.

### Hedgehog in malignancy

HH inhibition is an effective therapeutic approach in both basal cell carcinomas and meningiomas [29-31]. HH is an important tumor/stroma signaling component in several solid tumors including prostate, colon and pancreatic cancers [18, 32-35]. However, HH does not promote growth in all tumor models. Studies in bladder cancer suggest that HH:BMP tumor/stromal signaling restricts cancer growth [23]. Similarly, recent work in a pancreatic cancer model demonstrates a tumor growth inhibitory role for HH signaling with concurrent increases in tumor stromal elements [33]. Indeed, recent trials in colon and pancreatic cancer demonstrated no benefit and in one pancreatic trial, a possible trend toward harm with hedgehog inhibition [22, 36, 37]. In contrast, a HH inhibitor maintenance trial in ovarian cancer demonstrated no evidence of detriment [38] with a trend towards improvement in progression free survival. One potential explanation for the differences observed for HH inhibitors in ovarian cancer vs. colon, bladder, and pancreatic cancers is the developmental origin of the distinct tissues; embryologically, ovarian/fallopian tube epithelium is mesodermal in origin. In contrast pancreatic, colon, and bladder epithelial cells are endodermal in origin. During development, HH signaling has widely contrasting tissue-specific functions therefore it would not be surprising that HH signaling may act in a completely different manner in cells derived from different primary germ layers [39]. Further highlighting the complexity of HH signaling, we demonstrate that both tumor derived SHH and IHH form a signaling loop with CA-MSC-derived BMP4. Interestingly, *in vitro* SMO inhibition with IPI-926 more effectively disrupted the SHH/BMP4 loop as compared to the IHH/BMP4 loop. Additional work will be necessary to determine if this relates to currently undefined differences in SHH and IHH signaling or alternate pathways promoting tumor cell IHH secretion.

As noted above, HH inhibitors have not demonstrated significant clinical activity as single agents in ovarian cancer [38]. It has been suggested this trial failed as it did not select patients based on tumor HH expression [40]; the rate of HH activation in ovarian cancers (25-70%) remains controversial [19, 40, 41]. Our study suggests greatest potential clinical benefit in combining HH inhibition with chemotherapy. Indeed targeting HH may be a strategy to reverse platinum-resistance, an inevitable and deadly step in the progression of ovarian cancer.

### BMP4 and ovarian cancer

While a majority of the studies presented here, due to availability of clinically relevant inhibitors, focused on HH inhibition, BMP4 is also a critical component of this signaling loop. A BMP4 blocking antibody mimicked HH inhibition *in vitro*. Several studies have supported a critical role for BMP4 in ovarian cancer. BMP4 promotes migration and metastasis in ovarian cancer [42] and BMP4 stimulation of ovarian cancer cells can activate ID3 proto-oncogene expression [43]. We previously demonstrated, and confirmed here, that BMP signals from CA-MSCs increase the number of ALDH^+^ CSCs. We have also recently reported BMP2 as a regulator of CSC division increasing the proportion of ALDH^+^ cells [44]. Consistent with prior studies [45] [14], our data also demonstrate a modest increase in ALDH^+^ tumor cells after co-growth with normal adipose-derived MSCs (which at baseline have significantly lower levels of BMP4 compared to CA-MSCs). This may be due to alternate signaling pathways such as the IL6/CXCL7 loop reported in breast cancer [45]. Alternatively, this may be due to the development of a CA-MSC-like expression profile (with increasing levels of BMP2 and 4) in normal adipose-derived MSCs after long periods of tumor stimulation. Indeed, our previous work demonstrated tumor cell conditioning over time leads to changes in the overall expression profile of normal tissue MSCs yielding increases in BMP family proteins [14]. Further, IPI-926 blocks the normal MSC-mediated increase of ALDH^+^ tumor cells arguing for the development of a tumor:MSC HH:BMP signaling loop over time in normal MSCs.

While clearly important in ovarian cancer, effective clinical targeting of the BMP pathway has proven challenging. The identification of HH as a vital modulator of stromal BMP4 production provides a novel method to target BMP-associated tumorigenesis.

### MSCs and ovarian cancer

While controversy remains regarding the role of MSCs and cancer in general, numerous studies in ovarian cancer report a pro-tumorigenic role of MSCs. MSCs can directly impact the cancer cells to increase the growth of cancer stem cell-like cells [14], promote an epithelial mesenchymal transition [46], and increase resistance to apoptosis [47]. MSCs can also promote cancer cell chemotherapy resistance, even in the setting of hyperthermic therapy [48]. MSC are also reported to promote ovarian cancer invasive capacity and broadly impact the transcriptional profile of cancer cells to create a pro-metastatic phenotype [48, 49]. Interestingly, the impact of MSCs on cancer cells can be both via cytokine signaling as described here, and the exchange of cellular materials via exosomes [50, 51]. Similarly, MSCs can impact the host cells in the tumor microenvironment promoting ovarian cancer growth via increased angiogenesis [52] and suppression of the anti-tumor immune responses [53]. Taken together this data strongly supports MSCs as a therapeutic target in ovarian cancer. Given HH inhibition *in vivo* completely eliminated many of the pro-tumorigenic effects of CA-MSCs including increased angiogenesis, chemotherapy resistance, and increased tumor ‘stemness’ HH signaling may be a master regulator of CA-MSC function in ovarian cancer.

## CONCLUSION

In summary, the identification of a signaling loop with tumor derived HH and CA-MSC derived BMP4 adds further support to the importance of the tumor microenvironment and stromal signaling. Our findings help to elucidate the mechanism of CA-MSCs pro-tumorigenic functions. We demonstrate the critical importance of evaluating stromal targeted therapeutics in the presence of a human tumor stroma. Finally, these studies add to growing literature supporting the clinical use of HH inhibitors in ovarian cancer particularly in combination with chemotherapy and may specifically help revert platinum resistant disease to platinum sensitive disease.

## MATERIALS AND METHODS

### Tissue harvesting and culture

Patients samples were obtained in accordance with a protocol approved by the University of Michigan's IRB (IRB no HUM0009149). Tissue was processed for RNA isolation as previously described [54]. MSCs were isolated as previously described [14]. Briefly, to isolate CA-MSCs primary patient derived tumor tissue was plated in supplemented MEBM media and MSCs were selected for plastic adherence. CA-MSCs were characterized by cell surface marker expression (positive for CD105, CD90, CD73, CD44; negative for CD34, CD24, CD45) and their ability to differentiate into adipocytes, osteocytes or fibroblasts (following guidelines presented by The International Society for Cellular Therapy on the minimal criteria for defining multipotent mesenchymal stem cells[15]). A summary of CA-MSC cell surface marker expression and differentiation is provided in Supplemental Figure 6. Adipocyte and osteocyte differentiation was stimulated using differentiation media from StemCell technologies, (Vancover, BC). Fibroblast/myofibroblast differentiation was performed through prolonged indirect co-culture with ovarian tumor cells. Normal healthy donor-derived MSCs were purchased (for adipose MSCs) (Invitrogen, Grand Island, NY) or derived from normal omental or normal ovary surgical samples (using the same procedure as above for CA-MSC isolation from tumor tissue). Adipose, normal ovary and patient derived CA-MSCs were maintained in culture as previously described [14]. Mesenchymal stem cells were used at passage 8 or below. Ovarian cancer cell lines SKOV3, CAOV-3, COV318, Hey1 and PEO1 were obtained from ATCC and cultured in RPMI supplemented with 10% heat-inactivated FBS and 1% penicillin/streptomycin (SKOV3, Hey1) and DMEM supplemented with 10% heat-inactivated FBS and 1% penicillin/streptomycin (CAOV-3, COV318, PEO1).

### *Gli1*^*lacZ*^ mice

*Gli1*^*lacZ*^ reporter mice were treated for 5days with anti-mouse CD3e (clone 2c-11, Ebioscience San Diego, CA) antibody to reduce immune rejection of tumor cells. ID8 xenografts were created by injecting 10×10^6^ cells into the bilateral flanks of treated animals. ID8 xenograft tissue was obtained at time of sacrifice and B-Galactosidase staining was performed on tissues fixed in 4% PFA and embedded in OCT for cryosectioning [25]. Normal ovarian tissue was obtained from non-T cell depleted animals and processed as above. Quantification of B-Galactosidase staining was performed using ImageJ thresholding analysis [55]. Three independent sections of tumor and non-tumor stroma were used for quantification.

### Quantitative real-time PCR

RNA was isolated with the RNeasy Mini Kit (Qiagen, Hilden, Germany) and on-column DNase treatment (Qiagen, Hilden, Germany). RNA concentration was determined with NanoDrop ND-1000 Spectrophotometer. cDNA was synthesized with the SuperScript III First-Strand Synthesis System for RT-PCR (Invitrogen, Grand Island, NY) as previously described [56]. SYBR green-based RT-PCR was performed using the 7900HT Sequence Detection System (Applied Biosystems, Foster City, California) and respective primers. The comparative Ct method was used for data analysis, *HPRT* or *GAPDH* were used as the comparator genes.

### Immunoblotting

Portions of whole tumor or cell pellets were homogenized in RIPA buffer (Pierce, Rockford, IL) with complete protease inhibitor (Roche, Basel, Switzerland). Insoluble material was removed by centrifugation at 16,000g at 4°C for 15mins. Protein concentrations were determined using the Bradford Protein Assay Kit (Bio-Rad, Hercules, CA). Equal amounts of protein were separated on 4-12% NuPAGE SDS gel (Invitrogen, Grand Island, NY) and transferred onto a PVDF or nitrocellulose membrane. Antibodies for western blot analysis include anti-BMP4 (1:1000 dilution, Abcam, Cambridge, England), anti-SHH (1:500, 5E1, Developmental Studies Hybridoma Bank, Iowa City, IA), anti-Gli1 (1:500, PCRP-GLI1-1A1, Developmental Studies Hybridoma Bank, Iowa City, IA) and anti-B-actin (1:10,000 dilution, Sigma-Aldrich, St. Louis, MO). Bands were visualized using the ECL Kit (Pierce, Rockford, IL).

### *In vitro* SHH or TCM treatment of MSCs

Adipose, normal ovary and CA-MSCs plated at 1×10^5^ cells/6well in serum free supplemented MEBM media were treated with recombinant SHH (10-100ng/ml) (R&D systems, Pittsburg, PA) for 24hrs then processed for qRT-PCR as described above. For TCM, media was collected from tumor cell lines grown to 60% confluence after 3 days and centrifuged for 15min at 3500rpm to remove cellular debris. MSCs were treated with TCM ± IPI-926 (10nM) for 3 days then cells were processed for qRT-PCR or immunoblotting.

### *In vitro* BMP4 treatment of tumor cells

SKOV3, CAOV-3 and COV318 cells were plated (2×10^5^ cells/6 well dish) in serum-free media treated with 200ng/ml recombinant BMP4 (R&Dsystems, Pittsburg, PA) for 16 and 48hours. Cells were then processed for RT-PCR or immunoblotting.

### CA-MSC/tumor cell co-culture

SKOV3, CAOV-3, or COV318 were grown in co-culture with CA-MSCs using 24mm polystyrene transwell inserts, 0.4um pore (Sigma Aldrich, St. Louis, MO). CA-MSCs (5×10^4^cells) were seeded onto the top chamber in 1.5ml supplemented MEBM media and tumor cells (5×10^4^ cells) were seeded into the lower chamber in 2.5ml RPMI or DMEM ± 20nM IPI-926 or LDE225 (APExBIO) or the anti-BMP4 blocking antibody (R&D systems) for 5 days. CA-MSCs and tumor cells grown alone were used as controls.

### *In vivo* mouse MSC/tumor xenografts

Xenografts containing SKOV3 cells alone (1×10^6^ cells), SKOV3 + A-MSCs (0.5×10^6^ cells each) and SKOV3 + CA-MSCs (0.5×10^6^ cells each) with growth factor reduced matrigel (BD Biosciences, San Jose, CA) were injected into the bilateral axilla of NOD-SCID mice. After 5 days (to allow for tumor engraftment), half of the mice were treated with IPI-926 (Active Biochem, Hamburg, Germany) (20mg/kg daily via IP injection for 21 days as previously described by Lee et al. [57]). Mice were sacrificed when tumor burden exceeded 1500mm^3^. Tumor volume was calculated using the modified ellipsoid formula (L*W^2^)/2. Five mice with bilateral axillary xenografts were used per treatment group (*n* = 10 tumors per group). Two additional mice were injected with dsRed MSC/SKOV3 xenografts to serve as controls for FACS analysis below. NOD-SCID mice were maintained in accordance with institutional policies and all studies were performed with approval of the University Committee on Use and Care of Animals of the University of Michigan.

### *In vitro* chemotherapy resistance assays

CAOV-3, PEO1 or Hey1 cells labeled with GFP via lentiviral transduction were grown in co-culture with CA-MSCs in a 1:1 ratio with a total of 20,000 cells/24 well dish or in single culture with 20,000 cells/well and treated with cisplatin ± IPI-926 (20nM) for 24-48 hours. The number of viable GFP-tumor cells and CA-MSCs was analyzed via FACS using propidium iodide for viability stain and gating on GFP positive cells (for tumor cell analysis) or GFP negative cells (for CA-MSC analysis).

### *In vivo* chemotherapy resistance

Xenografts containing luciferase labeled CAOV-3 cells alone (1×10^6^ cells), CAOV-3/A-MSCs (0.5×10^6^ cells each) and CAOV-3/CA-MSCs (0.5×10^6^ cells each) with growth factor reduced matrigel were injected into the bilateral axilla of NOD-SCID mice. After 7 days, half of the mice were treated with cisplatin (1mg/kg weekly x 3 weeks). Bioluminescence was measured at day 40. Tumor volume measurement and euthanasia criteria were followed as above. Five mice with bilateral axillary xenografts were used per treatment group (*n* = 10 tumors per group).

The experiment was repeated with CAOV-3 cells alone and CAOV-3/CA-MSCs. After 5 days, half of the mice were treated with IPI-926 (20mg/kg daily via IP injection for 21 days). All mice received cisplatin treatment (1mg/kg weekly x 3 weeks starting on day 7 after injection). When the CAOV-3/CA-MSC cisplatin treated tumors reached ∼300mm3, half of the mice were retreated with cisplatin (1mg/kg weekly for up to 3 weeks) and half were retreated with cisplatin + IPI-926 (20mg/kg daily until time of sacrifice). Five mice with bilateral axillary xenografts were used per treatment group (*n* = 10 tumors per group) except in the CAOV-3/CA-MSC groups which contained 4 mice with bilateral tumors (*n* = 8 tumors).

### Immunohistochemistry

Tumors were cryosectioned or paraffin embedded. Paraffin-embedded tissue was H&E stained at the ULAM Pathology Cores for Animal Research at the University of Michigan and stroma quantified per low-power field (10x). Cryosectioned tumors were fixed and stained with anti-CD31 antibody (Dako) or permeablized and stained with anti-αSMA antibody- Cy3 (Abcam) and co-stained with mouse anti-human mitochondrial antibody (Life tech) with goat anti-mouse-alexa 488 secondary. CD31 + cells were counted to determine microvessel density per hpf (40x). Anti-αSMA staining was quantified by determining the area of positive staining per hpf (40x). 10 sections from 3 tumors per treatment group were analyzed [58].

### FACS

Xenograft single cell suspensions were analyzed for ALDH expressing cells with the Aldehyde Dehydrogenase-Based Cell Detection Kit as previously described [28] (Stemcell Technologies, Vancouver, Canada). FACS gating was based on live cells (via DAPI) with DEAB control for each sample. At least 3 independent tumors were used for each analysis except for the CAOV-3/CA-MSC+Cis+IPI-926 group in which two tumors were analyzed due to low tumor burden in this group. Flow cytometric analysis was performed on a BD Biosciences FACSCalibur through the University of Michigan Flow Cytometry Core.

### Statistics

All *in vitro* experiments were repeated independently at least three times with triplicate samples in each experiment unless otherwise stated. Statistical significance was evaluated using a 2-sided student's T-test. For all experiments, α = 0.05. For animal studies, *n* = 10 per group based on final tumor volume of control animals of ∼1000mm^3^ with an expected standard deviation of 30%. Non-linear regression analysis of tumor growth over time was performed for each group and curves compared to determine statistical significance. In all Figures, error bars represent standard error of the mean of composite values from independent experiments.

## SUPPLEMENTARY MATERIAL FIGURES


