# Supplementary material for: Human carcinoma-associated mesenchymal stem cells promote ovarian cancer chemotherapy resistance via a BMP4/HH signaling loop
Source: Oncotarget. 2016 Jan 9;7(6):6916–32. doi: 10.18632/oncotarget.6870 (PMC4872758; doi:10.18632/oncotarget.6870)
Supplement: Supplementary file 1 [file oncotarget-07-6916-s001.pdf]

Human carcinoma-associated mesenchymal stem cells promote ovarian cancer chemotherapy resistance *via* a BMP4/HH signaling loop

Supplementary Material

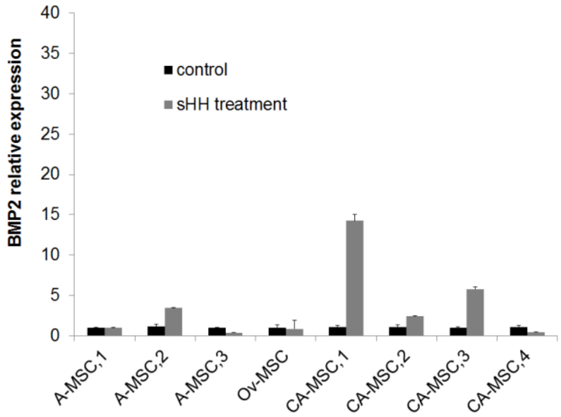

Supplemental Figure 1. BMP2 expression in response to SHH. qRT-PCR analysis of BMP2 expression in normal adipose MSCs (A-MSC1,2,3), normal ovary MSCs (Ov-MSC) and patient derived CA-MSCs (CA-MSC 1-4).

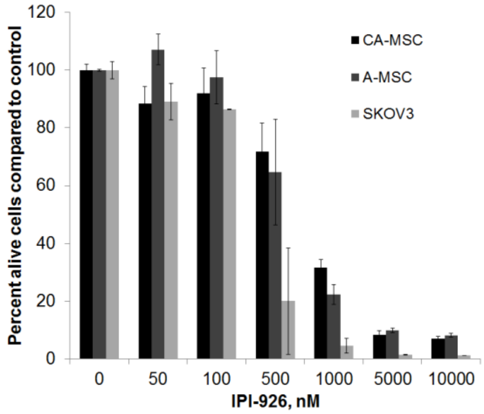

Supplemental Figure 2. IPI-926 does not affect MSC or tumor cell viability until drug concentrations exceed 100nM. Ovarian tumor cell line SKOV3, A-MSCs and CA-MSCs were treated for 72 hrs with IPI-926 at increasing concentrations and cell viability was assessed. Of note, 10-20nM IPI-926 was used for all other experiments.

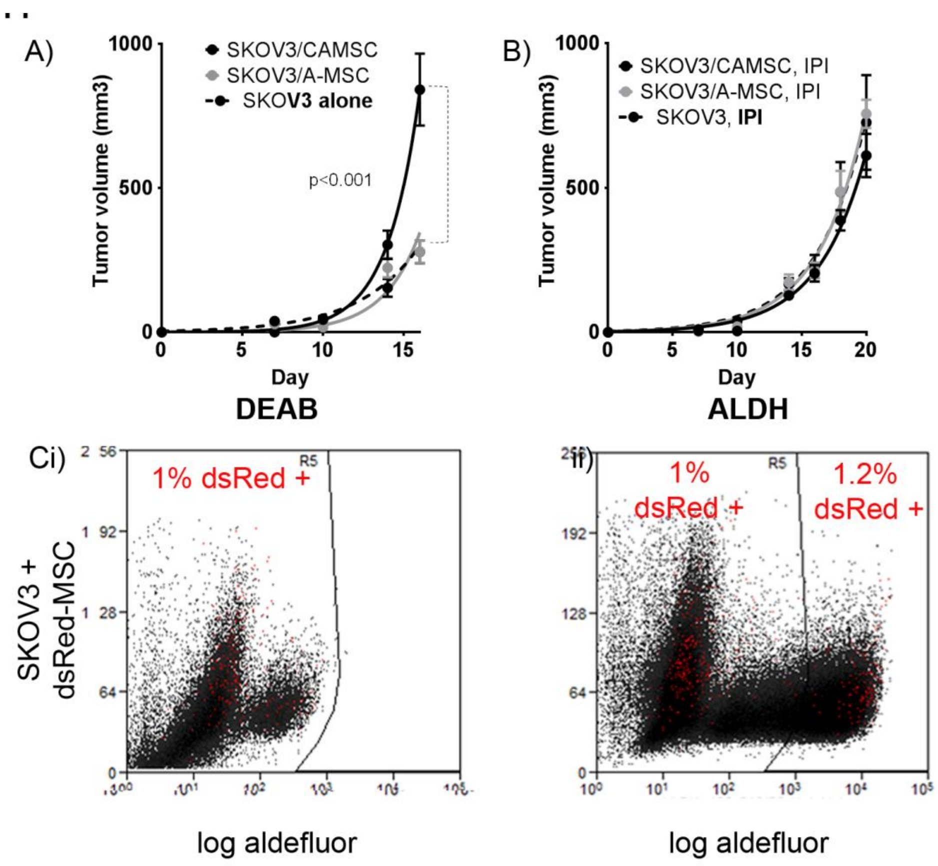

Supplemental Figure 3. Growth curves of tumors with MSCs ± IPI-926 and ALDH+ cells from tumors with dsRed labeled MSCs. A) Growth curves of tumors cells ± CA-MSCs or A-MSCs demonstrating growth advantage of CA-MSC-containing tumors B) Tumors treated with IPI-926 demonstrate IPI-926 blocks the CA-MSC-mediated increase in tumor growth. Ci) FACS plot demonstrating dsRed+/DEAB control, ii) dsRed+/ALDH- and dsRed+/ ALDH+ cells from SKOV3 + dsRed A-MSCs xenograft cell suspensions. dsRed labeled MSCs are depicted in Red.

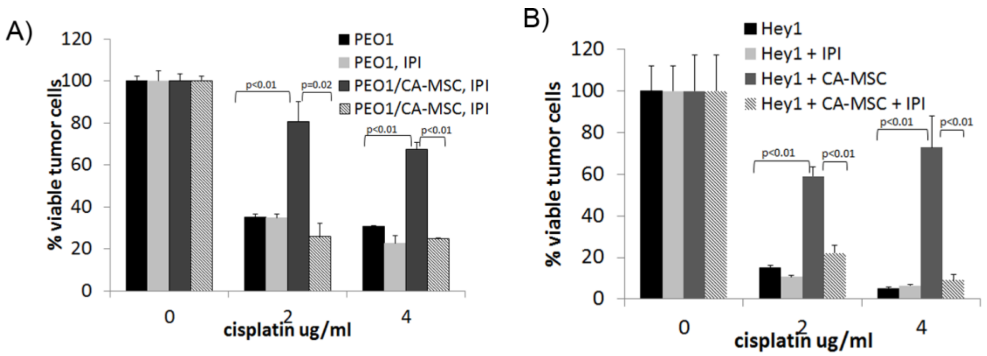

Supplemental Figure 4. HH inhibition blocks CA-MSC-mediated increases in in vitro chemotherapy resistance of high grade serous ovarian cancer cell lines. A) GFP-PEO-1 or B) GFP-Hey1 cells grown alone or with direct co-culture with CA-MSCs treated with increasing doses of cisplatin for 24hrs. Viable GFP-labeled cancer cells were quantified with FACs and normalized to 0ug/ml cisplatin group. Error bars=standard error of the mean from triplicate samples in one experiment.

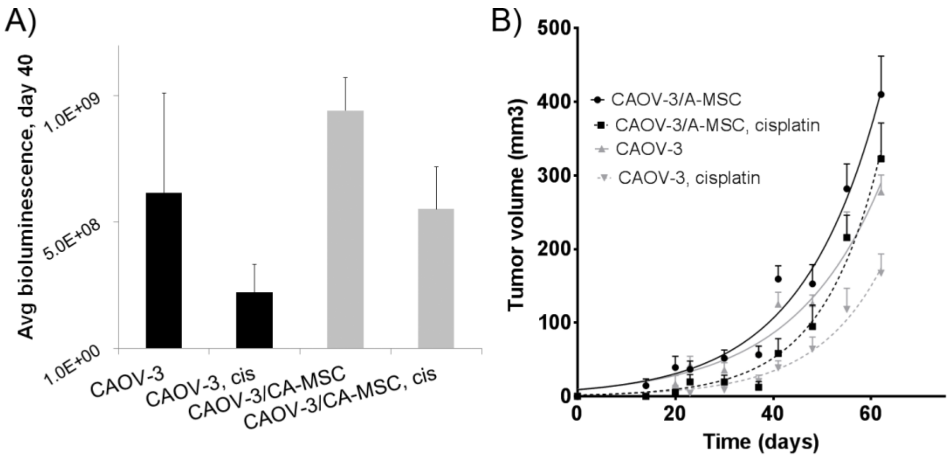

Supplemental Figure 5. Tumor bioluminescence and A-MSC tumor growth curves. A) Bioluminescence data at day 40 of CAOV-3 tumors ± CA-MSCs treated with and without cisplatin concordant with tumor growth curves. B) Tumor growth curves of CAOV-3 ± A-MSCs treated with and without cisplatin.

A)

	Patient sample CA-MSC (% positive)		
**Surface marker**	**Pt 324**	**Pt 370**	**Pt 437**
**CD105**	100	100	100
**CD90**	99	90	48
**CD73**	58	100	100
**CD44**	100	100	100
**CD45**	0	0	0
**CD34**	0.5	1	0.7
**CD24**	6.5	9	0.1

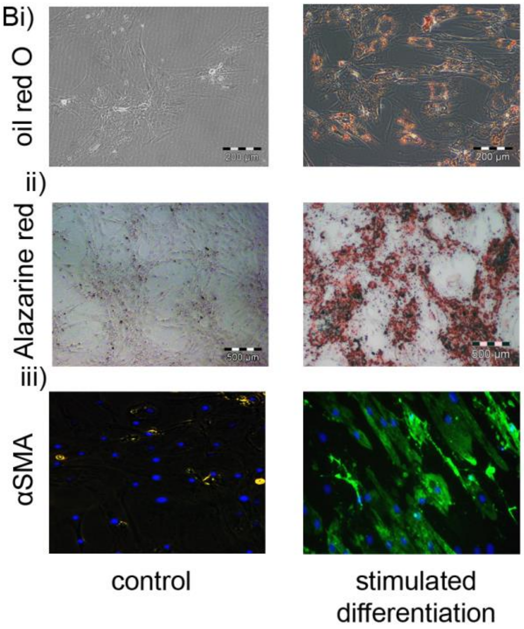

Supplemental Figure 6. CA-MSC characterization by cell surface markers and differentiation capacity. A) FACs analysis of CA-MSCs to determine percent positivity for cell surface makers characteristic of MSCs B) Representative pictures of CA-MSCs after stimulated differentiation into i) adipocytes (oil red O stain) ii) osteocytes (alazarine red stain) iii) myofibroblast (alpha smooth muscle actin (αSMA) stain).
